# Urea reduction ratio may be a simpler approach for measurement of adequacy of intermittent hemodialysis in acute kidney injury

**DOI:** 10.1186/s12882-019-1272-7

**Published:** 2019-03-06

**Authors:** Kelly V. Liang, Jane H. Zhang, Paul M. Palevsky

**Affiliations:** 1 Renal-Electrolyte Division, Department of Medicine, University of Pittsburgh School of Medicine, 3550 Terrace Street, Pittsburgh, PA 15213 USA; 2 Cooperative Studies Program Coordinating Center (151A), VA Connecticut Healthcare System, 950 Campbell Avenue, West Haven, CT 06516 USA; 3 Renal Section (111F-U) VA Pittsburgh Healthcare System, University Drive, Pittsburgh, PA 15240 USA

**Keywords:** Acute kidney injury, Renal replacement therapy, Adequacy, Kt/V, Urea reduction ratio

## Abstract

**Background:**

Assessment of adequacy of intermittent hemodialysis (IHD) is conventionally based upon urea kinetic models for calculation of single pool Kt/V_urea_ (Kt/V), with 1.2 accepted as minimum adequate clearance for thrice weekly IHD. In the Acute Renal Failure Trial Network (ATN) Study, adequacy of IHD in patients with acute kidney injury (AKI) was assessed using Kt/V. However, equations for Kt/V require volume of distribution of urea, which is highly variable in AKI. Therefore, simpler methods are needed to assess adequacy of IHD in AKI. We assessed correlation of urea reduction ratio (URR) with Kt/V and determined URR thresholds corresponding to Kt/V values to determine if URR could be a simpler means to assess the delivered dose of IHD.

**Methods:**

Using patients who received IHD for 2.5–6 h and with pre-dialysis BUN ≥20 mg/dL, we plotted URR against Kt/V. We determined URR thresholds (0.60 to 0.75) corresponding to Kt/V ≥ 1.2, 1.3, and 1.4. We generated receiver operating characteristic (ROC) curves for increasing URR values for each level of Kt/V to identify the corresponding thresholds of URR.

**Results:**

There was strong correlation between URR and Kt/V. ROC curves comparing URR with Kt/V ≥ 1.2, 1.3, and 1.4 had area under the curves (AUC) of 0.99. Sensitivity and specificity of URR ≥0.67 for corresponding values of Kt/V ≥ 1.2 were 0.769 (95% CI: 0.745 to 0.793) and 0.999 (95% CI: 0.997 to 1.000), respectively and the sensitivity and specificity of URR ≥0.67 for corresponding values of Kt/V ≥ 1.4 were 0.998 (95% CI: 0.995 to 1.000) and 0.791 (95% CI: 0.771 to 0.811), respectively.

**Conclusions:**

Targeting a URR ≥0.67 provides a simplified means of assessing adequacy of IHD in patients with AKI. Use of URR will enhance ability to assess delivery of small solute clearance and improve adherence with clinical practice guidelines in AKI.

## Background

Renal replacement therapy (RRT) is often required for the management of acute kidney injury (AKI). Depending on hemodynamic status, RRT may be provided by continuous dialysis (CRRT), hybrid forms of prolonged intermittent RRT (PIRRT) or intermittent dialysis (IHD). Assessment of adequacy of solute clearance in IHD is conventionally based upon urea kinetic models for calculation of single pool Kt/V_urea_ (Kt/V) [1–5]. The Kidney Disease: Improving Global Outcomes (KDIGO) Clinical Practice Guidelines for AKI recommend achieving a threshold Kt/V of at least 1.3 3x/week or 3.9/week when delivering IHD in the setting of AKI [6]. These recommendations were substantially based upon data from the Veterans Affairs/National Institutes of Health Acute Renal Failure Trial Network (ATN) study, a multicenter randomized trial comparing strategies of more-intensive and less-intensive RRT in critically ill patients with AKI which targeted a delivered Kt/V of 1.2–1.4 in patients receiving IHD [7].

Rigorous quantification of dialysis dose using urea kinetic modeling is based on assumptions related to net nitrogen balance and the generation and volume of distribution of urea that are not necessarily present in the critically ill patient with AKI [8, 9]. Patients with AKI are often hypercatabolic and in net negative nitrogen balance; may have rates of urea that are not constant over time; have alterations in regional blood flow, particularly in the setting of hemodynamic instability, that may produce disequilibrium in urea distribution between body compartments, invalidating standard single-pool models; and may have a volume of distribution of urea (V_urea_) that is variable and increased relative to patients with end-stage renal disease [5, 10, 11]. Thus, the applicability of the formal urea kinetic models and standard logarithmic estimating equations for calculation of Kt/V that were developed in the end-stage renal disease setting to estimate adequacy of small solute clearance in patients with AKI receiving IHD is questionable. In particular, uncertainty regarding adjustments for V_urea_ may introduce substantial error in the calculation of Kt/V using standard estimating equations.

In this post hoc analysis of data from the ATN study, we sought to determine if use of the urea reduction ratio (URR), based only on measurement of pre- and post-dialysis BUN and independent of estimates of V_urea_, would provide a simpler but sufficiently reliable method for assessment of the delivered small solute clearance during IHD for patients with AKI. In this analysis, we assessed the correlation between URR and the systematically calculated values of Kt/V in patients undergoing IHD in the ATN Study and determined URR thresholds that could be used as an alternative to Kt/V to assess small solute clearance in critically ill patients with AKI undergoing IHD.

## Methods

### ATN study design

In the ATN Study, 1124 critically ill adults with severe AKI attributable to acute tubular necrosis in the setting of sepsis or one or more failed non-renal organ systems were randomized to strategies of either less-intensive or more-intensive RRT. Details of the design and primary results of the ATN study have been previously published [7]. Written informed consent was obtained prior to enrollment and randomization of all participants. Participants randomized to more-intensive RRT (*N* = 563) received continuous venovenous hemodiafiltration (CVVHDF) at an effluent flow rate of 35 mL/kg/h or sustained low-efficiency dialysis (SLED) on a 6-day-per-week schedule (every day except Sunday) when hemodynamically unstable, or IHD on a 6-day-per-week schedule (every day except Sunday) when hemodynamically stable. Participants randomized to the less-intensive strategy (*N* = 561) received CVVHDF at an effluent flow rate of 20 mL/kg/h or SLED on a 3-day-per-week schedule (every other day except Sunday) when hemodynamically unstable, or IHD on a 3-day-per-week schedule (every other day except Sunday) when hemodynamically stable. In each treatment arm, IHD was prescribed with a targeted single-pool, variable-volume (spvv) Kt/V of 1.2–1.4 per treatment.

### Calculation of Kt/V

Kt/V was assessed at least three times per week during the first two weeks of study therapy and at least weekly thereafter using the second generation logarithmic (Daugirdas) equation:

$$ \mathrm{Kt}/\mathrm{V}=\hbox{-} \ln \left(\mathrm{R}\hbox{-} 0.008\ \mathrm{x}\ \mathrm{t}\right)+\left(4-3.5\ \mathrm{x}\ \mathrm{R}\right)\ \mathrm{x}\ 0.55\ \mathrm{x}\ \mathrm{UF}/{\mathrm{V}}_{\mathrm{urea}} $$

where *R* is the ratio of post-dialysis BUN (BUN_post_) divided by pre-dialysis BUN (BUN_pre_), *t* is the dialysis session duration in hours, *UF* is the ultrafiltration volume in liters, and *V*_*urea*_ is the estimated volume of distribution of urea [8, 9]. Blood samples for BUN were obtained immediately pre- and post-dialysis, with the post-dialysis BUN sample obtained using the slow-flow/stop-pump technique [14] to prevent sample dilution with recirculated blood and to minimize the variable effects of urea rebound. V_urea_ was initially estimated as 55% of pre-morbid body weight plus edema weight, where edema weight was calculated as pre-morbid body weight subtracted from current body weight and was recalculated each treatment. In patients with morbid obesity, defined as actual weight > 130% of ideal body weight (IBW), an adjusted pre-morbid body weight was calculated as IBW plus 25% of the difference between ideal and actual pre-morbid weight with IBW calculated based on height, gender, and frame size with adjustment for limb amputation [15]. The rationale for this correction is based on the reduction in the percentage body weight comprised by water (and hence V_urea_) as the percentage body fat increases.

### Calculation of urea reduction ratio (URR)

The urea reduction ratio was calculated as the quotient of the difference between the BUN_pre_ and the BUN_post_ divided by the BUN_pre_:

$$ \mathrm{URR}=\left({\mathrm{BUN}}_{\mathrm{pre}}-{\mathrm{BUN}}_{\mathrm{post}}\right)/{\mathrm{BUN}}_{\mathrm{pre}} $$

using the same BUN values used to calculate Kt/V.

### Statistical methods

We compared paired Kt/V and URR measurements from all IHD treatments with measured pre- and post-dialysis BUNs, excluding treatments that were < 2.5 h or > 6 h in duration. In addition, we excluded data from IHD treatments with pre-dialysis BUN values < 20 mg/dL as the reliability of URR and Kt/V calculations is markedly reduced when the pre-dialysis BUN is < 20 mg/dL. We report demographic and baseline clinical data as mean ± standard deviation, median (interquartile range) and number (percent) as appropriate. We examined the relationship between URR and Kt/V using linear and semilog plots and assessed the performance of URR against Kt/V thresholds of 1.2, 1.3 and 1.4 by generating receiver operating characteristic (ROC) curves and calculating the area under the curve (AUC) for each Kt/V value. We then identified values of URR that maximized sensitivity (fraction of measurements less than a given URR value when Kt/V was less than the target threshold) and specificity (fraction of measurements greater than a given URR value when Kt/V was greater than the target threshold) for Kt/V values of 1.2, 1.3 and 1.4.

## Results

### Number of Kt/V and URR measurements

Pre- and post-dialysis BUN measurements were obtained and Kt/V was calculated at least once during IHD treatments in 589 of the 1124 patients who participated in the ATN study. Characteristics of these patients are summarized in Table 1. There were a total of 2113 pre- and post-dialysis measurements in these 589 obtained during treatments with a duration between 2.5 and 6 h, a pre-dialysis BUN ≥20 mg/dL, and with documented pre- and post-dialysis weights to permit calculation of Kt/V. The mean number of Kt/V and URR measurements per patient was 3.6 ± 3.0.

**Table 1:** Characteristics of patients receiving intermittent hemodialysis (IHD) included in analysis

	Patients receiving IHD (*N* = 589)
Age (years)	64.3 ± 13.1
Gender	
Male	108 (18.4%)
Female	480 (81.6%)
Race or Ethnic Group	
White, non-Hispanic	477 (81.0%)
Black, non-Hispanic	80 (13.6%)
Hispanic	14 (2.4%)
Other	12 (2.0%)
Pre-morbid Weight (kg)	85.0 ± 18.8
Number of treatments with Kt/V measured	2113
Number of treatments with Kt/V measured per patient	
Mean ± SD	3.6 ± 3.0
Median (IQR)	3 (2–5)
Kt/V	
Mean ± SD	1.25 ± 0.35
Median (IQR)	1.25 (1.05–1.43)
URR	
Mean ± SD	0.64 ± 0.10
Median (IQR)	0.66 (059–0.71)

*IHD* = intermittent hemodialysis, *IQR* = interquartile range, *SD* = standard deviation, *URR* = urea reduction ratio

### Distribution of Kt/V and URR measurements

**Fig. 1:**
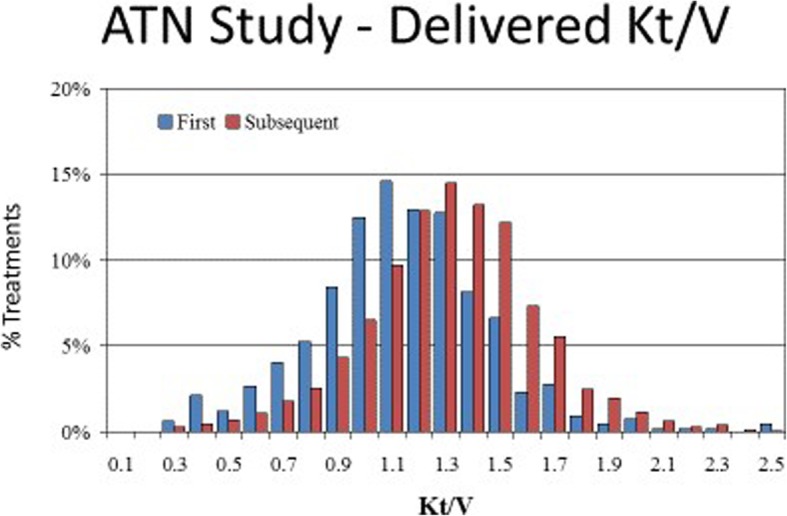
Distribution of Delivered Kt/V During Intermittent Hemodialysis in the ATN Trial. The overall mean ± standard deviation (SD) Kt/V for the first treatment was 1.13 ± 0.32 and for subsequent treatments was 1.32 ± 0.36, with a range of 0.3 to 2.5. The Kt/V values were normally distributed for both first and subsequent treatments

The overall mean ± standard deviation (SD) Kt/V for the first treatment was 1.13 ± 0.32 and for subsequent treatments was 1.32 ± 0.36, with a mean Kt/V of 1.25 ± 0.35 across all treatments. The Kt/V values were normally distributed for both first and subsequent treatments (Fig. 1). There were no significant differences between average Kt/V achieved for first or subsequent IHD treatments between the more intensive and less intensive RRT dose arms (data not shown). For this reason, all subsequent analyses were performed without stratification by treatment assignment.

### Relationship between URR and Kt/V

**Fig. 2:**
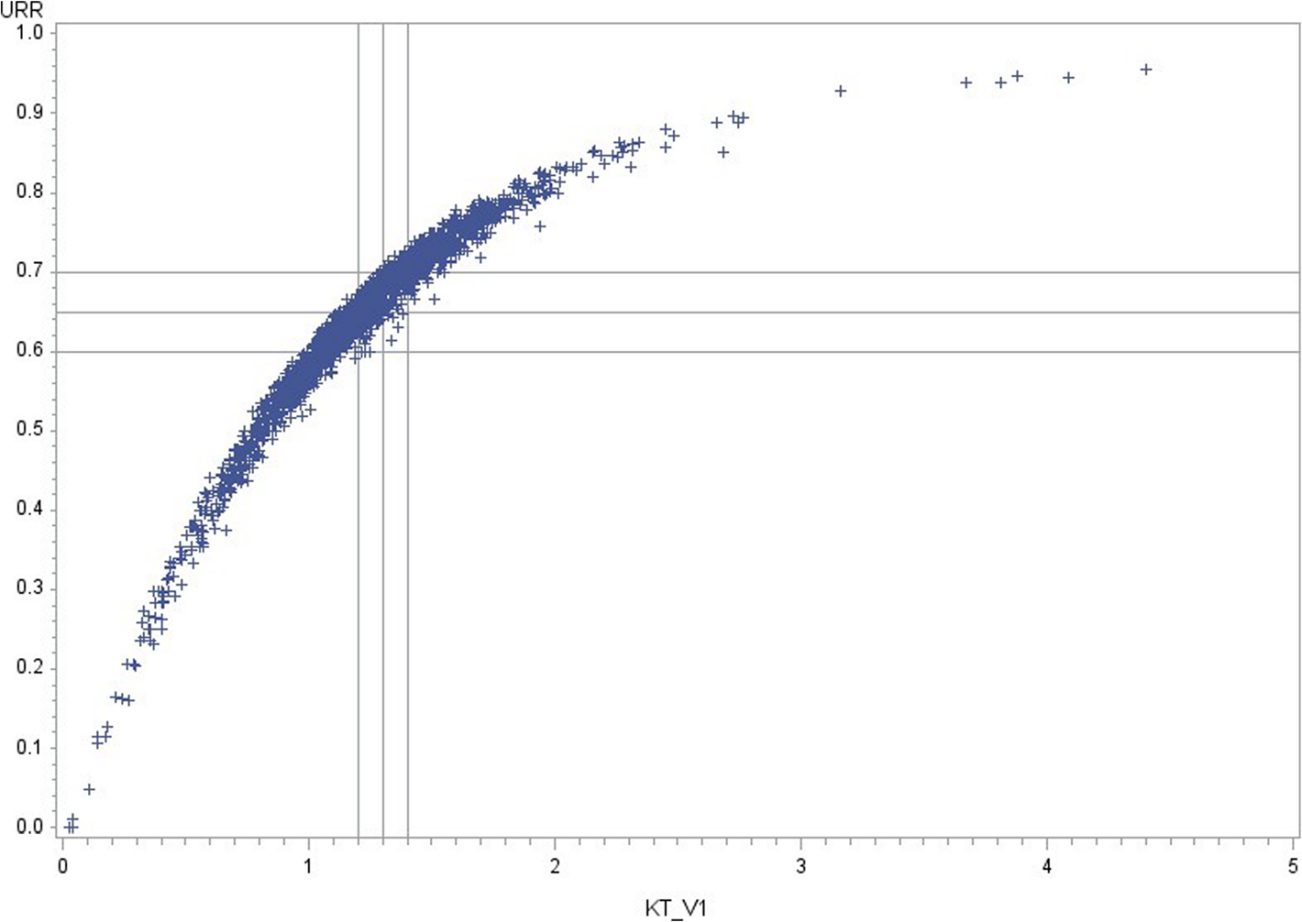
Plot of URR vs. Kt/V. There was a curvilinear relationship between URR and Kt/V

**Fig. 3:**
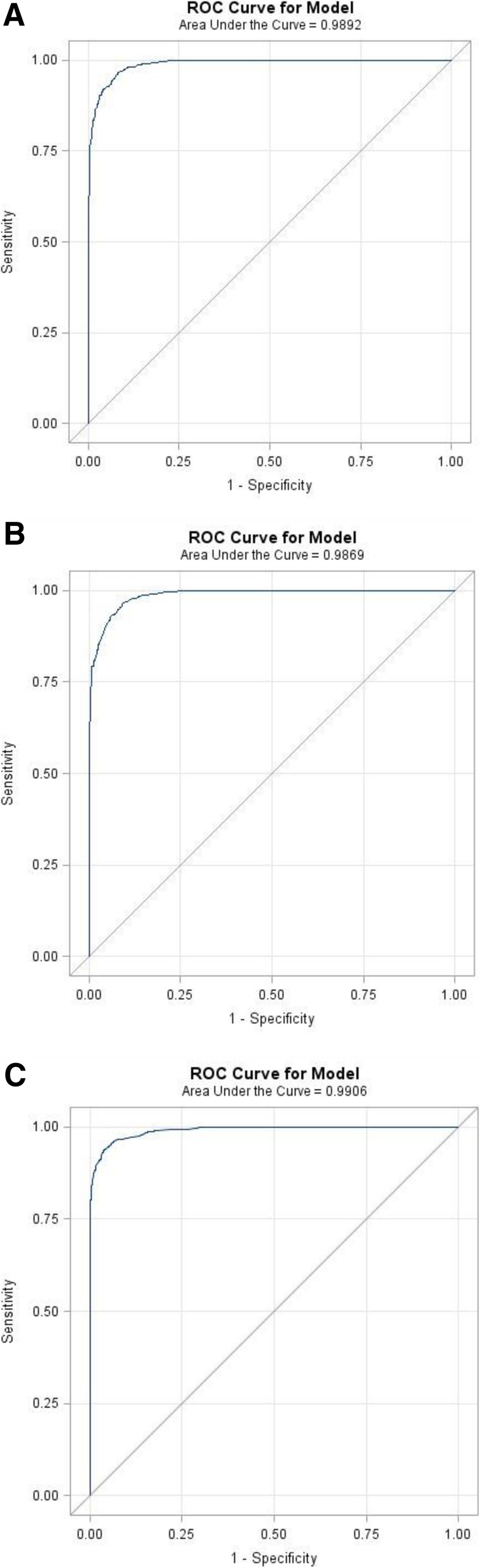
Receiver Operating Characteristic (ROC) Curves for Increasing Values of URR for Kt/V values ≥1.2, 1.3, and 1.4. **a**) Area under the curve (AUC) of the ROC curve for Kt/V ≥ 1.2 was 0.9892. **b**) AUC of the ROC curve for Kt/V ≥ 1.3 was 0.9869. **c**) AUC of the ROC curve for Kt/V ≥ 1.4 was 0.9906

There was a curvilinear relationship between URR and Kt/V (Fig. 2). ROC curves comparing URR with thresholds of Kt/V ≥ 1.2, 1.3, and 1.4 all had areas under the curves (AUC) of approximately 0.99 (Figs. 3a-c). The corresponding URR values for the range of Kt/V achieved in the ATN trial ranged from 0.60 to 0.75. The sensitivity and specificity for URR values between 0.60 and 0.75 for Kt/V thresholds of 1.2, 1.3 and 1.4 are provided in Table 2. The specificity for a Kt/V ≥ 1.2 was maximized when the URR was ≥0.67 (specificity 0.999; 95% CI: 0.997 to 1.000) and for a Kt/V ≥ 1.4 when the URR was ≥0.72 (specificity 0.995; 95% CI: 0.991 to 0.998). Similarly, the sensitivity for a Kt/V threshold of 1.4 was maximized at unity for URR values < 0.67.

**Table 2:** Sensitivity/specificity of URR for detecting Kt/V

URR	Kt/V ≥ 1.2		Kt/V ≥ 1.3		Kt/V ≥ 1.4	
	Sensitivity (95% CI)	Specificity (95% CI)	Sensitivity (95% CI)	Specificity (95% CI)	Sensitivity (95% CI)	Specificity (95% CI)
0.60	1.000 (1.000–1.000)	0.613 (0.582–0.643)	1.000 (1.000–1.000)	0.447 (0.421–0.474)	1.000 (1.000–1.000)	0.376 (0.352–0.400)
0.61	0.998 (0.995–1.000)	0.710 (0.681–0.738)	1.000 (1.000–1.000)	0.520 (0.494–0.547)	1.000 (1.000–1.000)	0.437 (0.413–0.462)
0.62	0.994 (0.989–0.998)	0.772 (0.746–0.799)	0.999 (0.997–1.000)	0.569 (0.543–0.596)	1.000 (1.000–1.000)	0.479 (0.455–0.504)
0.63	0.983 (0.976–0.990)	0.851 (0.829–0.873)	0.999 (0.997–1.000)	0.636 (0.610–0.662)	1.000 (1.000–1.000)	0.535 (0.511–0.560)
0.64	0.964 (0.954–0.975)	0.905 (0.887–0.924)	0.998 (0.995–1.000)	0.693 (0.668–0.717)	1.000 (1.000–1.000)	0.583 (0.559–0.608)
0.65	0.928 (0.914–0.943)	0.953 (0.939–0.97)	0.993 (0.988–0.999)	0.757 (0.734–0.780)	1.000 (1.000–1.000)	0.640 (0.617–0.664)
0.66	0.861 (0.842–0.881)	0.988 (0.981–0.995)	0.978 (0.968–0.988)	0.835 (0.815–0.855)	1.000 (1.000–1.000)	0.714 (0.691–0.736)
0.67	0.769 (0.745–0.793)	0.999 (0.997–1.000)	0.950 (0.936–0.965)	0.910 (0.894–0.925)	0.998 (0.995–1.000)	0.791 (0.771–0.811)
0.68	0.769 (0.745–0.793)	0.999 (0.997–1.000)	0.912 (0.893–0.931)	0.955 (0.944–0.966)	0.994 (0.987–1.000)	0.848 (0.831–0.866)
0.69	0.769 (0.745–0.793)	0.999 (0.997–1.000)	0.834 (0.809–0.859)	0.990 (0.984–0.995)	0.972 (0.959–0.985)	0.912 (0.898–0.926)
0.70	0.768 (0.745–0.793)	0.999 (0.997–1.000)	0.834 (0.809–0.859)	0.990 (0.984–0.995)	0.937 (0.917–0.956)	0.960 (0.950–0.969)
0.71	0.769 (0.745–0.793)	0.999 (0.997–1.000)	0.834 (0.809–0.859)	0.990 (0.984–0.995)	0.845 (0.817–0.874)	0.986 (0.980–0.992)
0.72	0.769 (0.745–0.793)	0.999 (0.997–1.000)	0.834 (0.809–0.859)	0.990 (0.984–0.995)	0.735 (0.700–0.770)	0.995 (0.991–0.998)
0.73	0.769 (0.745–0.793)	0.999 (0.997–1.000)	0.834 (0.809–0.859)	0.990 (0.984–0.995)	0.735 (0.700–0.770)	0.995 (0.991–0.998)
0.74	0.769 (0.745–0.793)	0.999 (0.997–1.000)	0.834 (0.809–0.859)	0.990 (0.984–0.995)	0.735 (0.700–0.770)	0.995 (0.991–0.998)
0.75	0.769 (0.745–0.793)	0.999 (0.997–1.000)	0.834 (0.809–0.859)	0.990 (0.984–0.995)	0.735 (0.700–0.770)	0.995 (0.991–0.998)

## Discussion

Our data demonstrate that there is a tight correlation between paired URR and Kt/V values and that measurement of URR provides a simpler method with adequate reliability for assessment of the delivered small solute clearance during intermittent hemodialysis in critically ill patients with AKI. This relationship is not unexpected, as the estimating equation used for calculating Kt/V is a function of the ratio between post-dialysis and pre-dialysis BUN, which is equal to 1 – URR [7, 8].

ROC curves evaluating the performance of URR relative to Kt/V thresholds of 1.2, 1.3, and 1.4 had AUC of > 0.99. Based on our data, a URR of ≥0.67 corresponded to a Kt/V ≥ 1.2 with > 99% accuracy with URR values of 0.67–0.72 corresponding to Kt/V values of 1.2–1.4. The KDIGO Clinical Practice Guidelines for AKI recommend delivery of Kt/V of at least 3.9 per week, corresponding to a Kt/V of > 1.3 on a thrice weekly dialysis schedule [6]. This would correspond to delivery of hemodialysis with a URR > 0.69 three times per week.

Measurement of adequacy of small solute clearance during intermittent hemodialysis is most rigorously based on formal urea kinetic modeling. However, many of the assumptions underlying these models in the stable outpatient with end-stage renal disease do not apply to critically ill patients with AKI [3, 16]. In particular, urea kinetic models assume the existence of a relative steady state during the modeling period, with the patient remaining in neutral nitrogen balance and the pre-dialysis state remaining relatively stable over repeated cycles of hemodialysis. These assumptions are often not valid in critically ill patients with AKI, the majority of whom are hypercatabolic and are in negative nitrogen balance or have variable rates of urea generation [13]. Furthermore, alterations in regional blood flow, particularly in patients who are hemodynamically unstable, may produce disequilibrium in urea distribution between body fluid compartments, invalidating standard single pool models [13]. In addition, unlike patients with end-stage renal disease on chronic hemodialysis, the volume of distribution of urea (V_urea_) in patients with AKI may exceed total body water [12] and may be highly variable due to variations in volume status over time. Thus, estimates of small solute kinetics in critically ill patients with AKI using formal urea kinetic models are inadequately validated as is the use of standard estimating equations [8, 9].

In the VA/NIH Acute Renal Failure Trial Network Study, Kt/V was estimated at least three times per week in patients receiving IHD during the first two weeks of study therapy and then at least weekly thereafter using a second generation logarithmic estimating equation. In order to adjust for variations in volume status, V_urea_ was re-estimated each treatment using an iterative process incorporating estimates of edema weight based on the difference between pre-morbid and post-dialysis body weight. The calculation of Kt/V used during the study was therefore cumbersome and not readily transferable to clinical practice. While the use of URR is less precise in estimating small solute clearance in intermittent renal replacement therapy, we have shown satisfactory correlation between paired values of URR and Kt/V in more than 3600 dialysis sessions. The use of URR is readily transferable to clinical practice and would facilitate assessment of the delivered dose of intermittent renal replacement therapy in the acute setting and the implementation of quality improvement processes to ensure appropriate delivery of therapy.

Limitations of our analysis must be noted. First and foremost, despite the fact that Kt/V calculation in the ATN study was rigorously protocolized, Kt/V was still calculated using a second generation logarithmic estimating equation rather than using formal urea kinetic modeling. Secondly, errors in estimation of Kt/V may have resulted from the protocolized methodology used in the ATN study to estimate V_urea_. Third, all dialysis treatments utilized catheters and catheter recirculation can both reduce the delivered dose of dialysis and interfere with ascertainment of post-dialysis BUN. However, recirculation will not affect the relationship between the measured Kt/V and URR. Furthermore, post-dialysis sampling during the ATN study was rigorously protocolized using standard slow-flow (blood pump at < 100 mL/min) or stop-pump sampling techniques to minimize the effect of catheter recirculation on ascertainment of the post-dialysis BUN concentration. Finally, since we relied on repeated measurements in individual patients, this may have introduced a degree of covariance between URR and Kt/V measurements that was not accounted for in our analytic approach. However, our data represents the largest dataset of rigorously measured pre- and post-dialysis BUNs and of systematically calculated estimates of Kt/V with a wide variance in both URR and Kt/V values. In addition, given the technical aspects of URR and Kt/V measurement, biological factors within individual patients are unlikely to have significantly contributed to covariance.

## Conclusions

In summary, we believe that the findings of our study validate the use of URR as a simpler means of assessing delivery of small solute clearance during intermittent hemodialysis in patients with AKI. Although this approach underestimates solute clearance associated with ultrafiltration, it should be recognized that multiple assumptions used in determination of Kt/V, such as the relationship of volume of distribution of urea to total body weight, do not hold in critically ill patients with AKI. The use of URR, which can be easily calculated, will enhance the ability to assess adequacy of acute dialysis treatments, improve adherence with the KDIGO clinical practice guidelines for delivered dose of intermittent renal replacement therapy in AKI, and facilitate implementation of quality assurance and performance improvement processes in the acute setting. Although the clearance of toxins is one of the key roles of dialysis in AKI, other important factors should also be considered when prescribing IHD in the critically ill population. Specifically, maintaining adequate fluid balance via fluid removal with dialysis, as well as considering the effect of more intensive hemodialysis on antibiotic clearance and efficacy of treatment of sepsis needs to be factored into decisions when prescribing IHD in AKI. Nevertheless, the need to achieve adequate small solute clearance remains a fundamental aspect in the prescription and monitoring of acute hemodialysis in critically ill patients with AKI. Our findings suggest that URR, which may be more simply calculated than Kt/V, is sufficient for assessment of delivery of small solute clearance. Our findings are of relevance to both clinical care and clinical research by simplifying the methods used to quantify small solute clearance during intermittent renal replacement therapy in critically ill patients with AKI.

## Abbreviations

AKI: acute kidney injury
ATN Study: Veterans Affairs/National Institutes of Health Acute Renal Failure Trial Network Study
AUC: Area under the curve
BUN: Blood urea nitrogen
IHD: Intermittent hemodialysis
KDIGO: Kidney Disease Improving Global Outcomes
PIRRT: Prolonged intermittent renal replacement therapy
ROC: Receiver operating characteristic curve
RRT: Renal replacement therapy
URR: Urea reduction ratio
VA: Veterans Affairs
